# Esophageal magnetic anastomosis for long gap congenital esophageal atresia: A case report

**DOI:** 10.1097/MD.0000000000042041

**Published:** 2025-04-04

**Authors:** Hefeng Wang, Tingzhen Yuan, Jinliang Zhang, Fengyu Gao, Lifeng Liu

**Affiliations:** a Department of Pediatric Surgery, Shandong Provincial Maternal and Child Health Care Hospital Affiliated to Qingdao University, Jinan, China; b School of Clinical Medicine, Shandong Second Medical University, Jinan, China; c Department of Hepatobiliary and Gastrointestinal Surgery, Shandong Provincial Maternal and Child Health Care Hospital Affiliated to Qingdao University, Jinan, China; d Department of Gastroenterology, Shandong Provincial Maternal and Child Health Care Hospital Affiliated to Qingdao University, Jinan, China.

**Keywords:** esophageal atresia, long gap esophageal atresia, magnamosis.

## Abstract

**Rationale:**

Long gap esophageal atresia (LGEA) is a rare congenital malformation. Magnamosis represents a minimally invasive technique for LGEA to achieve esophageal recanalization for feeding. Few cases had been reported worldwide.

**Patient concerns:**

An infant was diagnosed as congenital esophageal atresia by using prenatal ultrasound imaging including the absence of gastric bubble and polyhydramnios.

**Diagnoses:**

LGEA was confirmed after birth by esophagography through the gastrostomy and esophagus simultaneously.

**Interventions:**

Laparoscopic gastrostomy was performed on the 3rd day after birth to obtain nutrition. The esophagus was prolonged from the proximal and distal blind end weekly starting from 2 weeks after gastrostomy. Magnamosis was achieved with the help of thoracoscopy. The proximal and distal esophagus were fully separated and released under thoracoscopy. Two magnets were introduced into the proximal and distal esophageal pouch respectively. Chest X-rays were performed to demonstrate a progressive reduction of inter magnetic space. The esophageal imaging confirmed that the esophagus is connected, and the magnets were removed from mouth. An anastomotic leak was found, and the leak healed within 2 weeks.

**Outcomes:**

The infant achieved esophageal recanalization through magnetic anastomosis, allowing for oral feeding and maintained her native esophagus. Esophageal stenosis occurred at 4 weeks after magnetic anastomosis without other complications. Endoscopic balloon dilation was performed. The infant was followed up for 6 months, and exhibited durable esophageal patency with a good nutrition.

**Lessons:**

This result suggests that magnetic anastomosis is a feasible and effective treatment for LGEA in infants.

## 1. Introduction

Esophageal atresia (EA) characterized by the absence of continuity or narrowing of the esophagus with or without tracheal fistula is a rare congenital malformation.^[1]^ Long gap esophageal atresia (LGEA) defined as EA with no distal fistula is only a small portion (10%) in the EA spectrum.^[2]^ LGEA epitomizes the most challenging condition, unable to perform a primary esophageal anastomosis to bring the 2 esophageal ends together and restore continuity. Esophageal magnetic anastomosis (magnamosis) was recently proposed as a minimally invasive alternative option for LGEA infants.^[3–5]^ Magnamosis represents a nonsurgical minimally invasive technique for EA, especially for LGEA. Few cases had been reported worldwide.^[3–11]^ An infant with LGEA in our hospital was performed with esophageal magnetic anastomosis to achieve esophageal recanalization for feeding.

## 2. Case report

### 2.1. Diagnosis of LGEA

**Figure 1.**
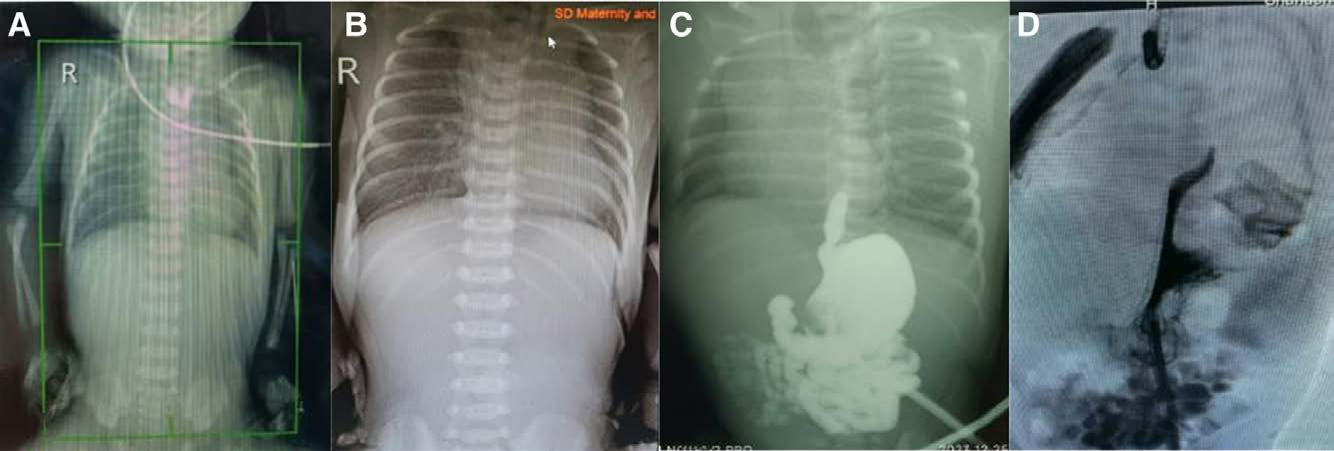
The diagnosis of long gap congenital esophageal atresia.

An infant was found to have congenital esophageal atresia during her mother’s pregnancy (24 weeks). No other abnormalities were found by using genetic test. Polyhydramnios was detected at 30 weeks gestation, and amniocentesis and decompression were performed at 34 weeks + 5 days. A girl weighing 2450 g was delivered at 37 weeks gestation. The infant vomited foam from the mouth, and the nasogastric catheter could not be inserted into the stomach, showing a “coiled-up” sign at the 10 cm. Esophageal imaging confirmed that the proximal end of the esophagus is blind and the blind end at the level of the 3rd thoracic vertebra (Fig. 1A). Abdominal X-ray shows no gas in the gastrointestinal tract of the abdomen (Fig. 1B). Laparoscopic gastrostomy was performed under general anesthesia with endotracheal intubation on the 3rd day after birth. Remote esophagography was performed intraoperation through the gastrostomy, revealing a blind distal end of the esophagus extending approximately 2 cm from the cardia to the chest cavity (Fig. 1C). A bronchos copy showed no tracheal fistula. Esophagography through the gastrostomy and esophagus simultaneously indicated that the EA was a long gap congenital esophageal atresia (Fig. 1D).

The study was approved by the local research ethics committee at the Shandong Provincial Maternal and Child Health Care Hospital Affiliated to Qingdao University according to the principle of the Helsinki Declaration II. Written informed consent from this participant was obtained.

## 3. Prolong of LGEA

**Figure 2.**
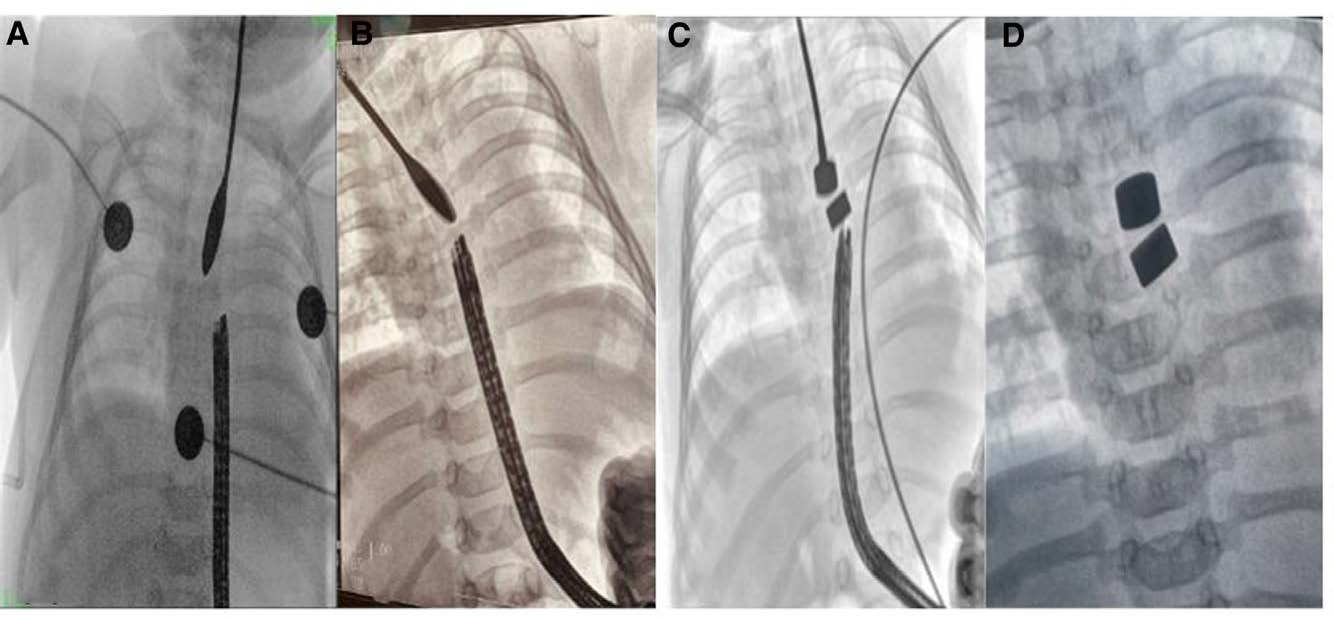
Prolong of LGEA under gastroscope. LGEA = long gap esophageal atresia.

Two weeks after gastrostomy, we performed gastroscopy examination to the child through the mouth and gastrostomy respectively under general anesthesia. The blind end of the proximal esophagus is about 10 cm away from the mouth, and the mucosa is smooth. The blind end of the distal esophagus is about 2 cm away from the cardia, and the mucosa is smooth. Starting from 2 weeks after gastrostomy, the esophagus was prolonged from the proximal and distal blind end weekly. Under gastroscope, a biliary probe was put into the upper pouch and pushed downwards, and a gastroscope was inserted into the lower esophagus through the gastrostomy and pushed upwards (Fig. 2A). The distance between the proximal and distal esophagus gradually approaches (Fig. 2B).

## 4. Magnetic anastomosis

**Figure 3.**
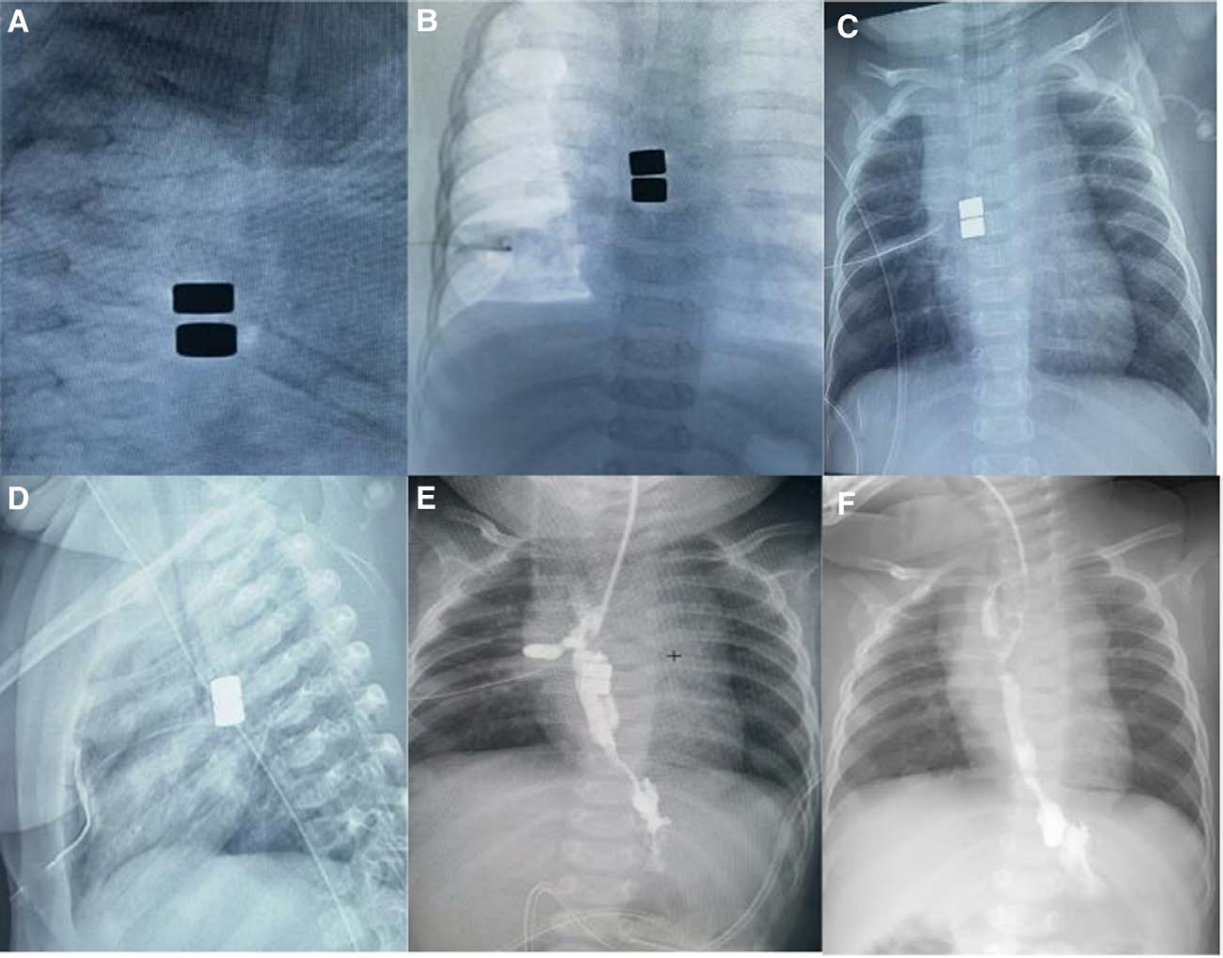
Magnetic anastomosis under thoracoscopy.

Cylindrical magnets with a diameter of 4 or 5 mm were used. These magnets are not specifically designed for medical use. We attempted to perform the magnetic anastomosis by using gastroscope. The 1st biliary probe-oriented magnet was orally introduced into upper pouch. Its twin magnet oriented by a gastroscopic biopsy forcep was introduced into distal esophageal pouch through the gastrostomy (Fig. 2C). The twin magnets might attract together (Fig. 2D), while the twin magnets were easily separated, and multiple attempts all failed. Finally, the proximal and distal esophagus were fully separated and released under thoracoscopy to bridge residual gap to achieve magnetic attraction. Two magnets were introduced into the proximal and distal esophageal pouch respectively. The twin magnets attracted together (Fig. 3A). Chest X-rays were performed to demonstrate a progressive reduction of inter magnetic space at the 3rd, 5th, and 7th days after operation. The 3rd day after operation, the space narrowed (Fig. 3B). The distance further decreased at the 5th days (Fig. 3C). The 7th day, close contact between magnets was achieved (Fig. 3D). The esophageal imaging confirmed that the esophagus is connected, while an anastomotic leak was found (Fig. 3E). Magnets were removed through mouth. The anastomotic leak was managed conservatively and healed within 2 weeks (Fig. 3F). During the period of magnetic anastomosis and anastomotic leak, the infant was fed with continuous enteral feeding via gastrostomy, and had a good nutrition.

## 5. Follow-up

The infant reached 6 months of postoperative follow-up. Esophageal stenosis occurred at 4 weeks after magnetic anastomosis without other complications. Endoscopic balloon dilation was performed. The child feeds orally now, with a good nutrition. The gastrostomy has been removed. The follow-up is still on.

## 6. Discussion

The LGEA infant achieved esophageal recanalization through magnetic anastomosis, allowing for oral feeding and maintained her native esophagus. The ultimate intention in the management of infant with LGEA is to enable esophageal continuity allowing for oral feeding. LGEA remains a technically challenging subset of EA, and there are currently no standardized guidelines for the treatment of LGEA.^[12,13]^ Surgical repairs involve either a primary anastomosis or replacing the esophagus with another autologous gastrointestinal conduit, namely stomach, jejunum, or colon.^[13,14]^ Complications such as esophageal leak or esophageal stenosis during surgical repair is relatively high. Magnamosis represents a nonsurgical minimally invasive technique for EA, especially for LGEA^[7]^ although some infants with wider gaps required surgery to allow magnamosis.^[3]^ The use of magnetic anastomosis for patients with EA was firstly reported in Argentina in 2009, where 5 infants was achieved.^[6]^ The magnets in the proximal and distal esophageal pouches attract each other due to their opposite polarity. The tissue between them becomes ischemic and sloughs, establishing the anastomosis. We attempted to achieve the magnetic anastomosis by using gastroscope, while the twin magnets separated easily. The reason might be that the gap and tension between the proximal and distal esophageal pouch exceed the strength of the magnetic field and fail to achieve attraction and connection. We achieved magnetic anastomosis with surgical assistance finally. Esophageal stenosis was the most common complications of magnetic anastomosis^[4,8,14,15]^, and subsequent esophageal dilations could expand the anastomosis and recreate the diameter of the esophagus. This infant occurred esophageal stenosis and endoscopic balloon dilation was performed subsequently. Esophageal leak is a rare complication,^[3,4,8]^ only 1 case report.^[5]^ The occurrence of esophageal leak in our case might due to lack of experience. The anastomotic leak healed well within following 2 weeks. In the future, magnetic anastomosis will even more exquisite.

A: Esophageal imaging confirmed that the proximal end of the esophagus is blind. B: Abdominal X-ray shows no gas in the gastrointestinal tract of the abdomen. C: Remote esophagography through the gastrostomy reveals a blind distal end of the esophagus. D: Esophagography through the gastrostomy and esophagus simultaneously indicated that the EA was a long gap congenital esophageal atresia.

A: A biliary probe was put into the upper pouch and pushed downwards, a gastroscope was inserted into the lower esophagus through the gastrostomy and pushed upwards. B: The distance between the proximal and distal esophagus gradually approaches after several prolongs. C: The magnets were introduced into proximal and distal esophagus respectively through the gastrostomy. D: The twin magnets attract together.

A: Two magnets were introduced into the proximal and distal esophageal pouch respectively, and the twin magnets attracted together. B: Chest X-rays display magnetic space narrowed at the 3rd day after operation. C: The distance further decreased at the 5th day after operation. D: Close contact was achieved at the 7th day. E: The esophageal imaging confirmed that the esophagus is connected, while an anastomotic leak was found. F: The anastomotic leak healed.

## Author contributions

**Data curation:** Hefeng Wang, Fengyu Gao, Lifeng Liu.

**Methodology:** Hefeng Wang, Jinliang Zhang, Fengyu Gao.

**Validation:** Hefeng Wang.

**Writing – original draft:** Tingzhen Yuan, Lifeng Liu.

**Writing – review & editing:** Hefeng Wang, Jinliang Zhang, Fengyu Gao, Lifeng Liu.
